# Survival Outcomes and Prognostic Factors of Metastatic Breast Cancer in Elderly Women: A Retrospective Study in Northeastern Morocco

**DOI:** 10.7759/cureus.91819

**Published:** 2025-09-08

**Authors:** Kaoutar Maadin, Hind Majd, Najlae Demnati Sadki, Mohammed Tarik Saoudi, Ouiame El Meliani, Lamiae Amaadour, Karima Oualla, Zineb Benbrahim, Nawfel Mellas, Samia Arifi

**Affiliations:** 1 Department of Medical Oncology, Faculty of Medicine, Pharmacy and Dental Medicine of Fez, University Sidi Mohamed Ben Abdellah, Hassan II University Hospital Center, Fez, MAR

**Keywords:** breast cancer, elderly women, metastatic, morocco, prognostic factors, survival, treatment

## Abstract

Introduction

Breast cancer (BC) is the most common malignancy among women worldwide and the leading cause of cancer-related mortality in women in Morocco. However, there is limited evidence on survival outcomes and treatment patterns among elderly patients with metastatic breast cancer (MBC) in this setting.

Methods

We conducted a retrospective cohort study at the Department of Medical Oncology, Hassan II University Hospital in Fez. The study included 101 female patients aged 65 years or older diagnosed with MBC between January 2012 and December 2016. The primary objectives were to estimate overall survival (OS) and progression-free survival (PFS). Secondary objectives included evaluating clinical characteristics, treatment outcomes, and prognostic factors.

Results

The median age of the cohort was 70.95 years (range: 65-88). De novo stage IV disease accounted for 61.4% (N = 62) of cases, while metastatic recurrence occurred in 38.6%. Among those with recurrence, 32.7% (N =33) had a relapse interval longer than 12 months. The median time to recurrence was 28 months (range: 1-246). The most common molecular subtypes were luminal B (38.6%; N = 38) and luminal A (29.7%; N = 30), followed by triple-negative (16.8%; N = 16) and HER2-positive (14.9%; N = 15). First-line treatments included chemotherapy (81.2%; N = 82), hormone therapy (32.7%; N = 33), anti-HER2 targeted therapy (16.8%; N = 17), and best supportive care (3%; N = 3). Median OS was 56 months (95% CI: 39.21-72.78), and median PFS was 42 months (95% CI: 27.7-56.2). The estimated three- and five-year OS rates were 64.2% and 55%, and the PFS rates were 59.8% and 51.2%, respectively. Univariate and multivariate analyses identified increasing tumour size, liver metastases, de novo metastatic disease, and a relapse interval of less than 12 months as significant poor prognostic factors for both OS and PFS. The presence of visceral metastases or combined visceral and bone metastases was associated with worse OS only. By contrast, hormone therapy was significantly associated with improved survival.

Conclusions

This study highlights the clinical and therapeutic characteristics of elderly patients with MBC in Morocco. Tumour size, relapse interval, and liver metastases were key prognostic factors for OS and PFS, while hormone therapy was associated with better survival outcomes.

## Introduction

Breast cancer (BC) represents a significant global health challenge. It is the most diagnosed cancer worldwide, with an estimated 2.26 million cases reported in 2020, and remains the leading cause of cancer-related mortality among women. Although historically considered a disease predominantly affecting developed countries, more than half of all BC diagnoses and two-thirds of BC-related deaths occurred in less developed regions of the world in 2020 [1]. The 2016 report from the Greater Casablanca Cancer Registry in Morocco identified BC as the most prevalent malignancy among Moroccan women [2].

The term “elderly” can be defined both chronologically and functionally. While its chronological definition varies across the literature [3], it is conventionally applied to individuals aged 65 years or older. Within this population, those aged 65 to 74 are often referred to as the “early elderly” and those aged 75 and above as the “late elderly." The expanding elderly population has been accompanied by a corresponding rise in the incidence of BC diagnoses among older women [4,5]. There is strong evidence that both the incidence and mortality rates of BC increase with age. According to the National Cancer Institute’s Surveillance, Epidemiology, and End Results (SEER) Cancer Statistics Review, 43% of newly diagnosed BC patients are aged ≥65 years, and 20% are aged ≥75 years. The majority of BC-related deaths also occur in these age groups [6]. Regardless of age, metastatic breast cancer (MBC) remains an incurable condition, with approximately 25% of patients surviving five years after diagnosis [7]. These statistics underscore the importance of screening and early detection.

Treatment guidelines for older women with MBC remain poorly defined. Management decisions often rely on physicians’ clinical experience due to the underrepresentation of elderly patients in clinical trials [8-10]. Moreover, the elderly population exhibits considerable variability in physical, cognitive, and psychosocial functioning. Some individuals, as well as younger patients, may tolerate chemotherapy, while others experience unpredictable and severe toxicities [11]. Therefore, treatment strategies for elderly patients should be individualized, guided by geriatric assessment tools that require further validation through prospective clinical trials [12,13].

In Morocco and other resource-limited settings, little is known about the survival outcomes and prognostic factors of elderly women with MBC, as this population is often underrepresented in clinical trials and research.

The primary objective of this study was to assess survival outcomes, including overall survival (OS) and progression-free survival (PFS), in elderly women with MBC. The secondary objectives were to describe clinical and pathological characteristics and treatment patterns and to identify prognostic factors influencing survival in this underrepresented population.

By providing real-world data from Morocco, this study may help guide treatment decisions and improve the management of elderly women with MBC in similar resource-constrained environments.

## Materials and methods

Study design and participants

This retrospective, descriptive, and analytical cohort study was conducted at the Department of Medical Oncology of the University Hospital Centre of Fez. A total of 283 patients diagnosed with BC between January 2012 and December 2016 were included. Among them, 101 patients with MBC were selected for analysis. Eligible participants were women aged 65 years or older, diagnosed with either de novo or recurrent MBC. Patients with incomplete or insufficient medical records were systematically excluded from the analysis to ensure data reliability and replicability. Participants were divided into three age groups at the time of MBC diagnosis: 65-69 years, 70-74 years, and ≥75 years. Comorbidities and performance status were also assessed. Clinical data included histological subtypes, TNM stage, SBR grading, metastatic status (de novo or relapsed), and the recurrence-free interval (≤12 months or >12 months). Metastatic sites were grouped into four categories: lung/pleura, liver, bone, and lymph nodes. Four immunohistochemical subtypes were identified: luminal A, luminal B, HER2-positive, and triple-negative (TN). Regarding treatment, patients were categorized into four groups: those who received hormonal therapy, chemotherapy, targeted therapy (anti-HER2), or palliative care. Treatment response was assessed and categorized as disease stability, partial or complete response, or disease progression.

Statistical analysis

In the initial step, descriptive statistical analysis was performed. Qualitative variables were described in percentages, while quantitative data were presented as mean ± standard deviation. Associations between categorical variables and treatment outcomes were assessed using the chi-square test. The significance threshold was set at p < 0.05. OS and PFS were estimated using the Kaplan-Meier method, and survival curves were compared with the log-rank test. Prognostic factors were identified through univariate and multivariate Cox regression analysis. All statistical analyses were done using Microsoft Excel (Microsoft Corp., USA) and IBM SPSS Statistics for Windows, Version 23.0 (released 2015, IBM Corp., Armonk, NY).

## Results

Patients’ characteristics

The median age of the cohort was 70.95 years (range: 65-88 years), with 44.6% (N = 45) of patients aged 65-69 years, 36.6% (N = 37) aged 70-74 years, and 18.8% (N = 19) over 75 years. Most patients (80.2%; N = 81) had an Eastern Cooperative Oncology Group (ECOG) performance status of 0-1, and 38.7% (N = 39) had comorbidities, mainly hypertension, diabetes, and cardiovascular disease. Invasive ductal carcinoma (IDC) was the predominant histological type (91.1%; N = 92). At diagnosis, 41.6% (N = 42) of patients were classified as cT4, with 58.3% (N = 59) presenting mobile lymph node metastases. All patients had metastatic disease. Grade II SBR was the most common histological grade (58.4%; N = 59). De novo stage IV cases represented 61.4% (N = 62) of the population. Metastatic recurrences accounted for 38.6% (N =39) of patients, with 6% (n= 6) experiencing recurrence within 12 months and 32.7% (N = 33) after more than 12 months. The median time to recurrence was 28 months (range: 1-246 months). The most common metastatic sites were pleuropulmonary (75.2%; N =76), bone (56.4%; N = 57), liver (32.7%; N = 33), and lymph nodes (31.7%; N = 32). A total of 25.7% (N = 26) of patients had more than three metastatic sites, while 74.3% (N = 75) had fewer than three. Bone-only metastases were found in 10% (N = 10) of patients, visceral-only in 44.6% (N = 45), and both visceral and bone metastases in 45.5% (N = 46). Molecular profiling showed that the most frequent subtypes were luminal B (38.6%; N = 39) and luminal A (29.7%; N = 30), followed by triple-negative (16.8%; N = 16) and HER2-positive (14.9%; N = 15) (Table 1).

**Table 1:** Descriptive characteristics of elderly patients with metastatic breast cancer. Values are expressed as number and percentage (N (%)). IDC-NOS: invasive ductal carcinoma not otherwise specified, BCS: breast-conserving surgery

Characteristics		Number of patients (N = 101)	Frequency (%)
Age	Group 65-69	45	44.6%
	Group 70-74	37	36.6%
	Group ≥75	19	18.8%
Comorbidities	Hypertension	14	13.9%
	Diabetes	12	11.9%
	Cardiopathy	4	4.0%
	Other comorbidities	9	8.9%
Revealing signs	Extension assessment	88	87.1%
	Respiratory symptoms	7	6.9%
	Bone symptoms	2	2.0%
	Other	4	4.0%
Histological type	IDC-NOS	92	91.1%
	Other	9	8.9%
Tumor size	cT1	12	11.9%
	cT2	31	30.7%
	cT3	16	15.8%
	cT4(a.b.c.d)	42	41.6%
Lymph node stage	N0	42	41.6%
	N1	37	36.6%
	N2	16	15.8%
	N3	6	5.9%
SBR Grade	I	10	9.9%
	II	56	58.4%
	III	32	31.7%
Immunohistochemical	Luminal A	30	29.7%
	Luminal B	39	38.6%
	Triple negative	16	16.8%
	HER2+	15	14.9%
Disease status	De novo	62	61.4%
	Relapse	39	38.6%
Relapse interval	<12 months	6	5.9%
	>12 months	33	32.7%
Metastasis sites	Lung and pleural	76	75.2%
	Bone	57	56.4%
	Liver	33	32.7%
	Lymph node	32	31.7%
Metastasis sites	Visceral	45	44.6%
	Bone	10	9.9%
	Both	46	45.5%
Number of metastatic sites	Metastases <3	75	74.3%
	Metastases ≥3	26	25.7%
Treatment	Hormone therapy	33	32.7%
	Chemotherapy	82	81.2%
	Anti-HER2 therapy	17	16.8%
	BCS	3	3%

Treatment patterns

First-line treatment included chemotherapy in 81.2% (N = 82) of patients, consisting mainly of anthracyclines combined with cyclophosphamide or taxanes. Hormonal therapy was administered to 32.7% (N = 33) of patients. Anti-HER2 targeted therapy combined with chemotherapy was initiated in 16.8% (N = 17) of cases. Optimal supportive care (best supportive care, BSC), aimed at symptom relief and patient comfort, was provided in 3% of patients (N = 3). Most treatment-related toxicities were grades 1-2 and consisted mainly of hematologic events such as neutropenia and peripheral neuropathy. No cases of cardiac toxicity were reported. Among the 101 patients, 15.8% (N= 16) had stable disease, 5% (N = 5) achieved a partial response, and 7.9% (N = 8) were lost to follow-up. However, disease progression was observed in 71.3% (N = 72) of cases. Regarding patient outcomes, 65.3% (N = 66) of patients died during follow-up, 28.7% (N = 29) were lost to follow-up, and 5.9% (N = 6) were still alive (Table 2).

**Table 2:** Response to treatment and evolution of metastatic breast cancer in elderly patients. Values are expressed as number and percentage (N (%)).

		Number of patients (N = 101)	Frequency (%)
Response to treatment	Stable disease	16	15.8%
	Partial response	5	5.0%
	Progression	72	71.3%
	Lost to follow-up	8	7.9%
Evolution	Deceased	66	65.3%
	Alive	6	5.9%
	Lost to follow-up	29	28.7%

Univariate analysis using the chi-square test showed significant associations: hormone therapy was significantly associated with bone metastases, chemotherapy was more frequently used in the younger elderly group, and anti-HER2 therapy was associated with larger tumours. In addition, univariate analysis showed that patient outcomes were significantly influenced by the presence of liver metastases, relapse interval, response to treatment, and metastatic site (p < 0.05) (Table 3).

**Table 3:** Results of the univariate analysis of treatments and patient status by different variables. p-values < 0.05 are according to the chi-square test.

Variables studied		Chi-square value	Ddl	P-value
Hormone therapy	Bone metastases	5.291	1	0.021
Chemotherapy	Age group	11.287	2	0.004
Anti-HER2	Tumor size (cT1-cT4)	8.531	3	0.036
Status of patients	Relapse interval	13.085	4	0.011
	Site of metastasis	22.652	4	<0.001
	Response to treatment	15.192	6	0.019
	Liver metastasis	8.904	2	0.012

Survival

**Figure 1:**
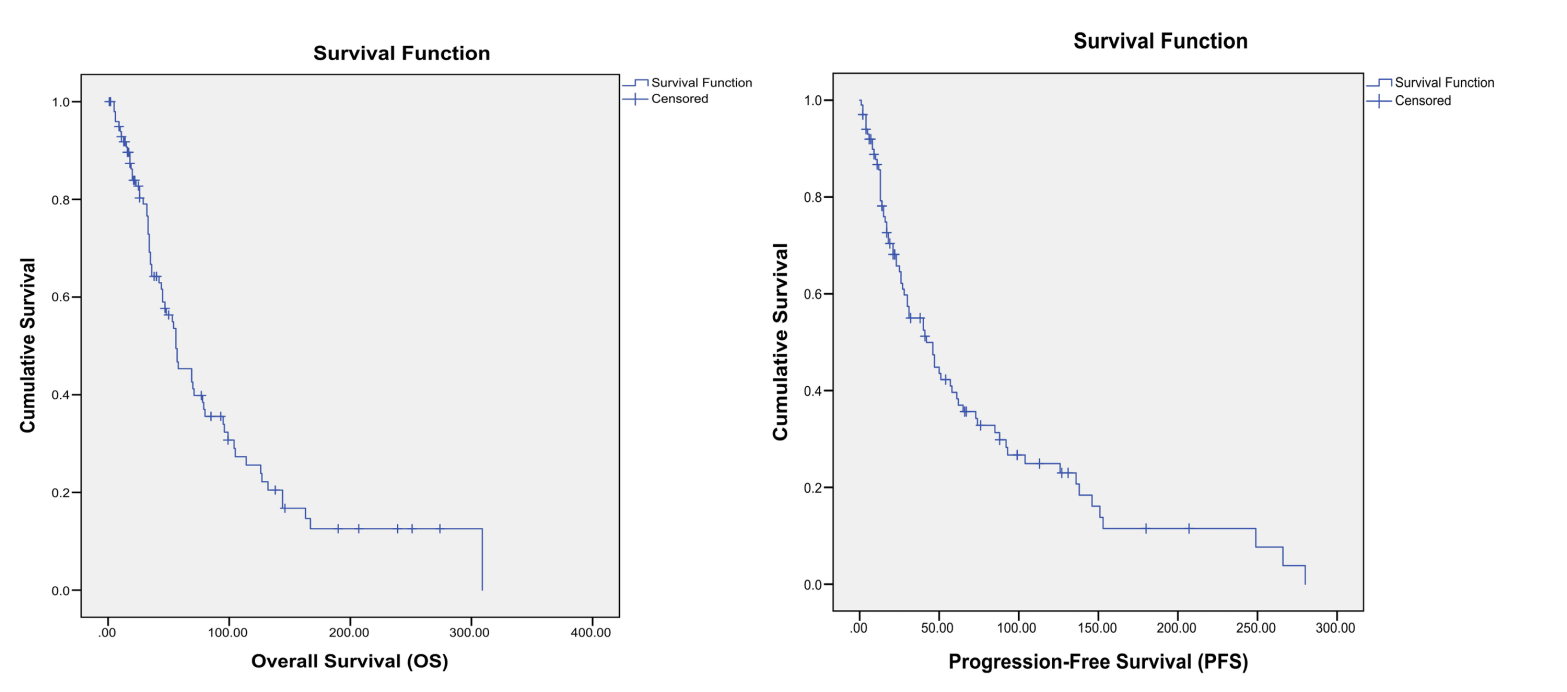
Kaplan–Meier curves showing OS probability and PFS probability. OS: overall survival, PFS: progression-free survival

After a median follow-up of 40 months (range: 1-305 months), the median OS was 56 months (95% CI: 39.21-72.78), and the median PFS was 42 months (95% CI: 27.7-56.2) (Figure 1). The estimated three-year and five-year OS rates were 64.2% and 55%, respectively, while the three-year and five-year PFS rates were 59.8% and 51.2%, respectively.

**Figure 2:**
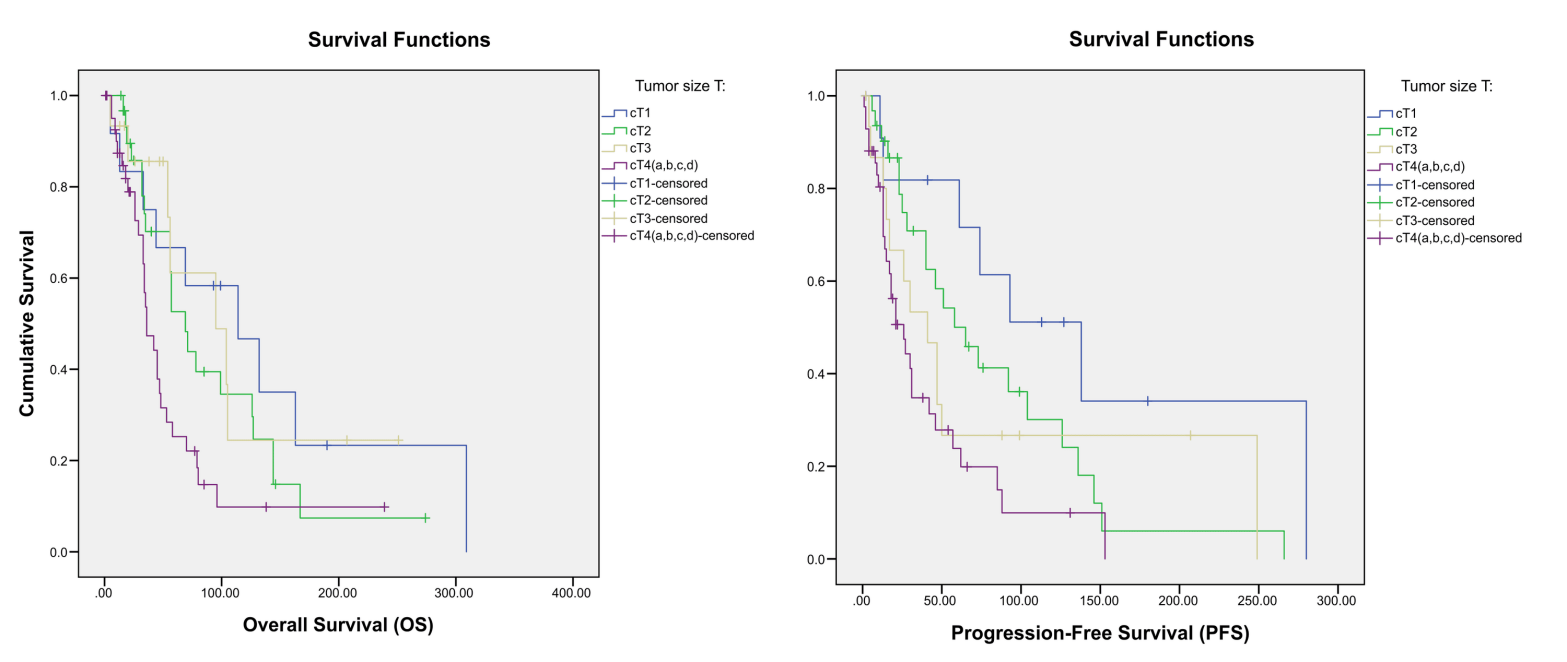
Kaplan–Meier curves showing the overall survival (OS) and progression-free survival (PFS) according to the size of the tumor. p-values < 0.05 are according to the log-rank test.

**Figure 3:**
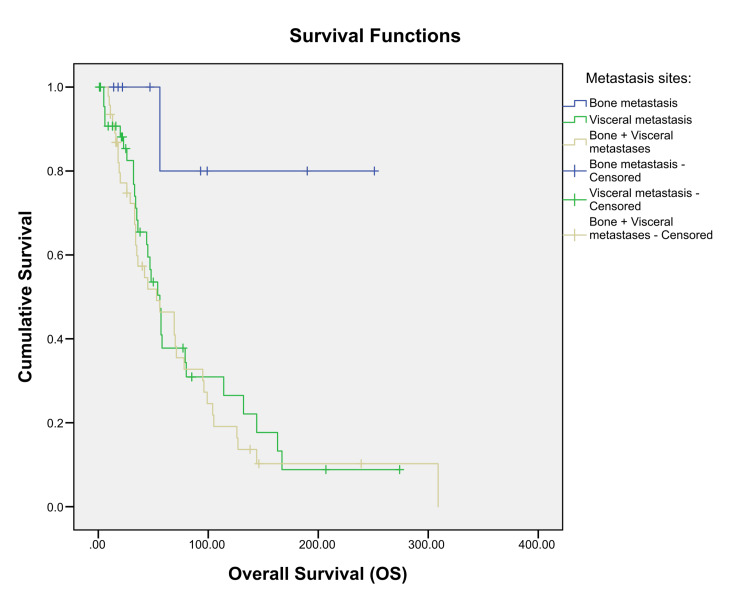
Kaplan-Meier curve showing overall survival according to the site of metastasis. p-values < 0.05 are according to the log-rank test.

**Figure 4:**
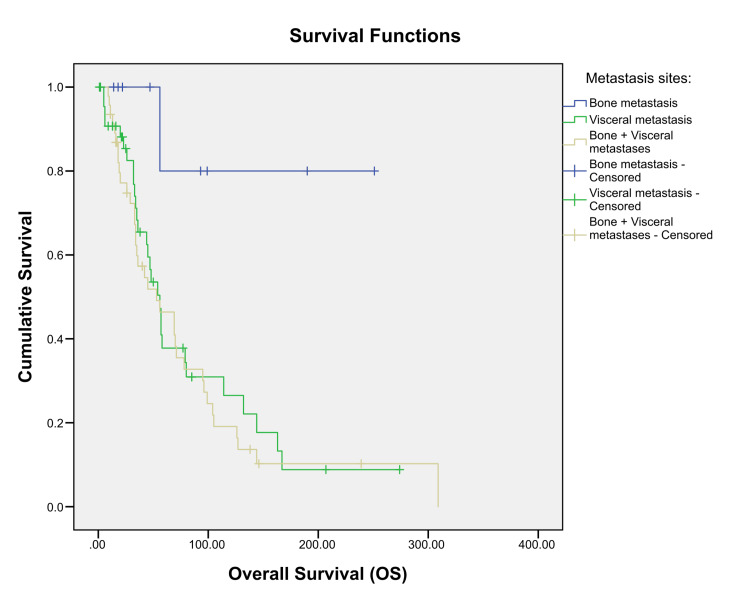
Kaplan-Meier curve showing overall survival according to the site of metastasis. p-values < 0.05 are according to the log-rank test.

**Figure 5:**
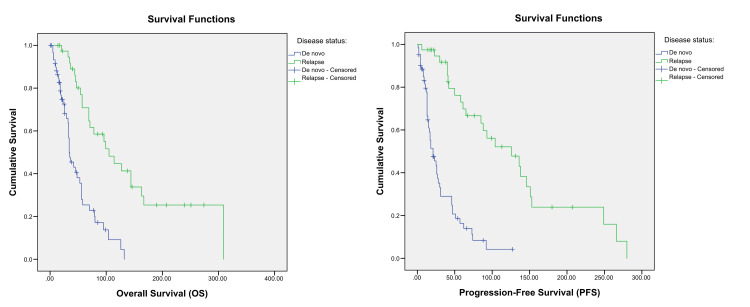
Kaplan–Meier curves showing the overall survival and progression-free survival according to the initial disease status. p-values < 0.05 are according to the log-rank test.

**Figure 6:**
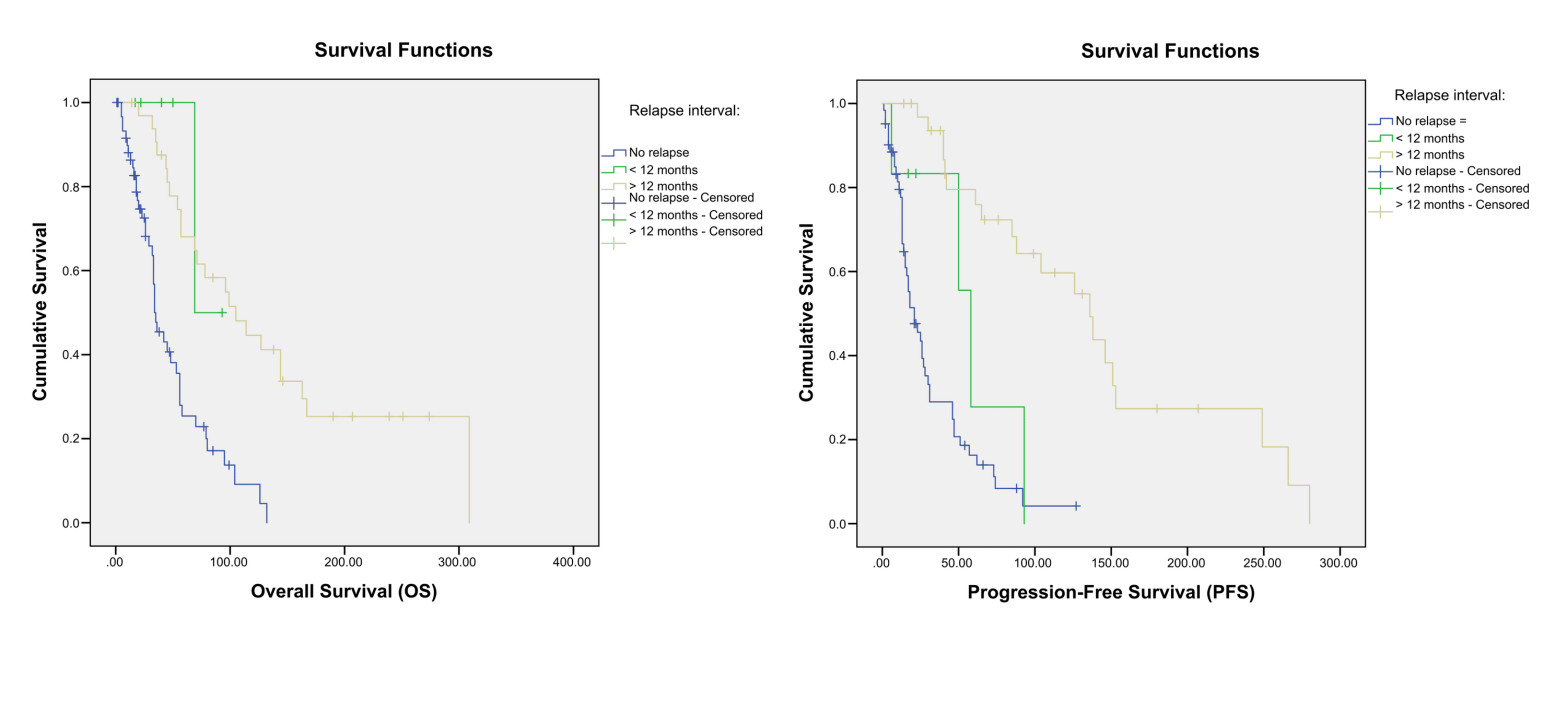
Kaplan–Meier curves showing the overall survival and progression-free survival according to the relapse interval. p-values < 0.05 are according to the log-rank test.

**Figure 7:**
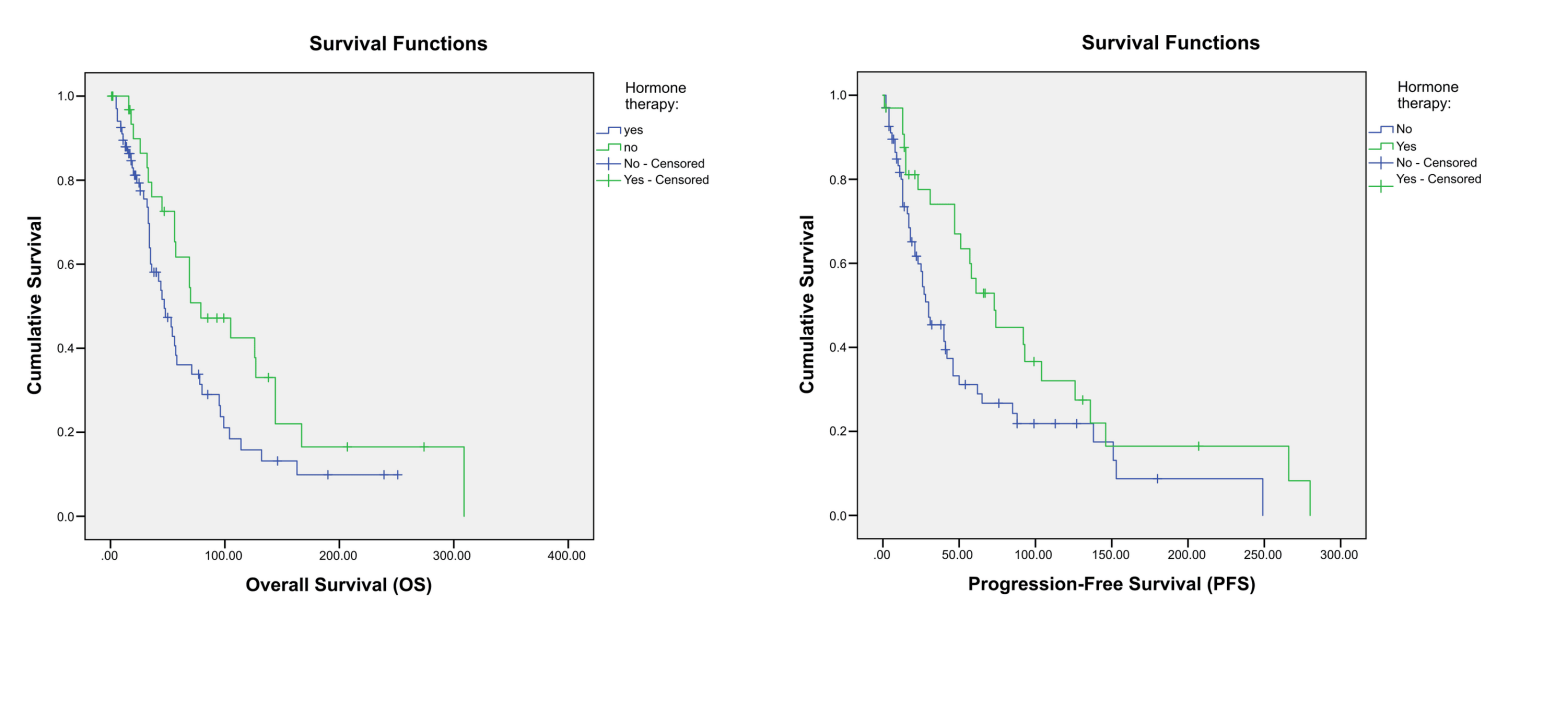
Kaplan–Meier curves showing the overall survival and progression-free survival according to hormonal therapy. p-values < 0.05 are according to the log-rank test.

Univariate Cox regression analysis identified several poor prognostic factors for both OS and PFS, including increased tumour size (Figure 2), presence of liver metastases (Figure 3), de novo MBC (Figure 5), and a relapse interval of less than 12 months (Figure 6) (p < 0.05). The presence of visceral metastases or combined visceral and bone metastases was associated with significantly poorer OS only (p < 0.05) (Figure 4). Conversely, hormone therapy, a relapse interval longer than 12 months, and relapsed (as opposed to de novo) MBC were significantly associated with improved survival outcomes (p < 0.05) (Figure 7, Table 4).

**Table 4:** Results of the univariate Cox regression prognostic factor analysis for OS (overall survival) and PFS (progression-free survival). p < 0.05 were calculated using the Cox regression test.

		OS: median					PFS: median				
		Estimate	Standard error	Confidence interval 95 %		P-value	Estimate	Standard error	Confidence interval 95 %		P-value
				Lower limit	Upper limit				Lower limit	Upper limit	
Tumor size	cT1	114	41.699	32.27	195.73	0.15	138	40.938	57.761	218.239	0.005
	cT2	69	8.695	51.957	86.043		65	15.771	34.088	95.912	
	cT3	95	33.183	29.961	160.039		41	10.144	21.118	60.882	
	cT4	36	5.516	25.188	46.812		26	6.197	13.854	38.146	
	Global	56	8.563	39.216	72.784		42	7.265	27.76	56.24	
Hepatic metastasis	No	78	11.375	55.705	100.295	0.001	50	9.331	31.71	68.29	0.015
	Yes	35	5.71	23.808	46.192		27	6.858	13.559	40.441	
	Global	56	8.563	39.216	72.784		42	7.265	27.76	56.24	
Metastasis site	Bone Metastasis	.	.	.	.	0.027	74	29.64	15.906	132.094	0.452
	Visceral metastasis	56	5.454	45.31	66.69		42	8.818	24.716	59.284	
	bone and visceral metastasis	53	15.936	21.766	84.234		31	10.363	10.689	51.311	
	Global	56	8.563	39.216	72.784		42	7.265	27.76	56.24	
Initial disease status	de novo	34	4.758	24.674	43.326	<0.001	21	4.618	11.948	30.052	<0.001
	Relapse	105	15.468	74.683	135.317		126	30.626	65.974	186.026	
	Global	56	8.563	39.216	72.784		42	7.265	27.76	56.24	
Relapse interval	No Relapse	34	4.758	24.674	43.326	<0.001	21	4.618	11.948	30.052	<0.001
	< 12 months	69	.	.	.		58	6.693	44.881	71.119	
	> 12 months	105	23.77	58.412	151.588		136	22.19	92.508	179.492	
	Global	56	8.563	39.216	72.784		42	7.265	27.76	56.24	
Hormonal therapy	No	47	6.332	34.589	59.411	0.044	30	5.876	18.482	41.518	0.03
	Yes	79	23.519	32.903	125.097		73	12.877	47.761	98.239	
	Global	56	8.563	39.216	72.784		42	7.265	27.76	56.24	

Multivariate Cox regression analysis confirmed that the larger tumour size and the presence of metastases were independent poor prognostic factors for OS, while liver metastases, short relapse interval, and the absence of hormone therapy were independent predictors of poorer PFS (p < 0.05) (Table 5).

**Table 5:** Results of multivariate Cox regression prognostic factor analysis for OS (overall survival) and PFS (progression-free survival). p < 0.05 were calculated using the Cox regression test.

	Prognostic factors	B	Error standard	Wald	Ddl	Sig.	Exp(B)	95.0% CI for Exp(B)	
								Lower limit	Upper limit
OS	Tumor size	0.331	0.121	7.484	1	0.006	1.392	1.098	1.764
	Hepatic metastasis	0.89	0.256	12.031	1	0.001	2.434	1.472	4.024
PFS	Hepatic metastasis	0.814	0.266	9.397	1	0.002	2.258	1.341	3.8
	Relapse interval	-1.022	0.17	36.149	1	<0.001	0.36	0.258	0.502
	Hormonal therapy	-0.644	0.265	5.893	1	0.015	0.525	0.312	0.883

## Discussion

This retrospective analysis of elderly Moroccan women with MBC provides valuable insights into the clinical characteristics, treatment patterns, and survival outcomes of a frequently underrepresented population. The median age was 70.95 years (range: 65-88), with two-thirds of patients classified as younger elderly (<75 years). Most had a good performance status (ECOG 0-1), and fewer than half presented with significant comorbidities, supporting the notion that chronological age alone should not preclude active oncologic treatment. This aligns with current geriatric oncology recommendations, which emphasize individualized therapeutic strategies rather than age-based decisions [14].

The predominance of invasive ductal carcinoma and SBR grade II tumours is consistent with established epidemiological trends [15]. The molecular subtype distribution in our cohort was led by luminal B and luminal A, followed by triple-negative and HER2-positive subtypes. Luminal subtypes are more common in older women and are generally associated with more favourable prognoses [16,17]. The most frequent metastatic sites were pleuropulmonary, followed by the bone and liver, which aligns with the known metastatic patterns of luminal tumours. Of note, 45.5% of patients had both visceral and bone metastases, a combination that has been linked to poor outcomes [18,19].

Chemotherapy emerged as the most common first-line treatment, particularly among patients under 75 years of age [16]. This choice may be attributed to several factors: the high proportion of patients with good ECOG status (0-1 in 80% of cases), the aggressiveness of the disease (61% had de novo MBC), the frequent involvement of both visceral and bone sites, and the limited availability of CDK4/6 inhibitors at the time, compounded by issues related to treatment reimbursement and social coverage. However, hormone therapy was significantly associated with improved survival outcomes. The treatment was well tolerated with manageable grade 1 to 2 toxicities. These findings highlight the importance of basing therapeutic decisions on clinical status rather than age alone, in line with international geriatric oncology guidelines [20].

In our study, the median OS and PFS were 56 months (95% CI: 39.21-72.78) and 42 months (95% CI: 27.7-56.2), respectively. The estimated three-year and five-year OS rates were 64.2% and 55%, while the PFS rates were 59.8% and 51.2%, respectively. These outcomes are lower than those reported in other studies, where the median OS often ranges from 86.6% to 89.7% [21]. This difference may be partly explained by the high prevalence of visceral metastases in our cohort.

Univariate Cox regression analysis identified several poor prognostic factors for both OS and PFS, including increased tumour size, presence of liver metastases, de novo MBC, and a relapse interval of less than 12 months. The presence of visceral metastases or combined visceral and bone involvement was significantly associated with worse OS (p < 0.05), confirming the detrimental impact of multi-site and visceral spread. These findings are in agreement with previous studies highlighting visceral involvement, particularly in the liver, and high tumour burden as strong negative predictors of survival [22]. Interestingly, patients with de novo metastatic disease had poorer outcomes than those with relapsed disease, which contrasts with some previous reports suggesting better survival in de novo MBC. This discrepancy may reflect more aggressive tumour biology or delayed diagnosis in our setting [23]. In contrast, hormone therapy, a relapse interval longer than 12 months, and relapsed disease were significantly associated with improved survival outcomes, consistent with findings from earlier studies [24,25].

Multivariate Cox regression analysis confirmed these associations: tumour size and the presence of metastases were independent prognostic factors for OS, while liver metastases, shorter relapse interval, and absence of hormone therapy were independent predictors of poorer PFS. These results reinforce the prognostic importance of metastatic burden, timing of recurrence, and therapeutic strategies-findings that are consistent with the broader literature.

A major strength of this study lies in its long median follow-up duration (50 months), which provides a more comprehensive perspective on long-term survival in this population. It also offers real-world data describing the clinical profile, therapeutic approaches, survival outcomes, and prognostic factors in elderly patients with MBC.

While certain limitations should be acknowledged, they reflect common challenges encountered in routine clinical practice, particularly in resource-limited settings. The relatively high proportion of patients lost to follow-up may have influenced survival outcomes, potentially underestimating or biasing survival estimates. In addition, the absence of systematic geriatric assessment limited our ability to fully capture the heterogeneity of functional and cognitive status in this elderly population, thereby reducing the generalizability of our findings. Finally, disparities in access to targeted therapies, such as anti-HER2 agents and CDK4/6 inhibitors, may have impacted treatment strategies and outcomes. Future prospective studies are necessary to validate and expand upon our findings.

## Conclusions

This study provides valuable insights into the clinical characteristics, treatment patterns, and prognostic factors of MBC in elderly women in a Moroccan context. Despite the advanced age of the population, active treatment, particularly chemotherapy and hormone therapy, was frequently administered, supporting the feasibility and relevance of individualized treatment approaches beyond chronological age. Survival outcomes were relatively favourable, with a median overall survival of 56 months. Independent poor prognostic factors included larger tumour size, the presence of liver metastases, a short relapse interval, and the absence of hormone therapy. These findings highlight the importance of early diagnosis, accurate assessment of metastatic burden, and the use of molecular subtype-guided treatment in this vulnerable population. However, the retrospective and single-center design, together with the proportion of patients lost to follow-up, may limit the generalizability of our findings and should be considered when interpreting survival outcomes. In resource-limited settings, improving access to endocrine and targeted therapies, enhancing follow-up systems, and incorporating geriatric assessment tools into clinical decision-making are key priorities. Further prospective studies are warranted to validate these findings and to optimize the management of elderly women with MBC.
